# A Dual Bioconjugated Virus-Like Nanoparticle as a Drug Delivery System and Comparison with a pH-Responsive Delivery System

**DOI:** 10.3390/nano8040236

**Published:** 2018-04-13

**Authors:** Roya Biabanikhankahdani, Kok Lian Ho, Noorjahan Banu Alitheen, Wen Siang Tan

**Affiliations:** 1 Department of Microbiology, Faculty of Biotechnology and Biomolecular Sciences, Universiti Putra Malaysia, 43400 UPM Serdang, Selangor, Malaysia; roya_biabani@yahoo.com; 2 Department of Pathology, Faculty of Medicine and Health Sciences, Universiti Putra Malaysia, 43400 UPM Serdang, Selangor, Malaysia; klho@upm.edu.my; 3 Department of Cell and Molecular Biology, Faculty of Biotechnology and Biomolecular Sciences, Universiti Putra Malaysia, 43400 UPM Serdang, Selangor, Malaysia; noorjahan@upm.edu.my; 4 Institute of Bioscience, Universiti Putra Malaysia, 43400 UPM Serdang, Selangor, Malaysia

**Keywords:** virus-like particle, drug delivery, conjugation, cancer, nanocarrier

## Abstract

Modifications of virus-like nanoparticles (VLNPs) using chemical conjugation techniques have brought the field of virology closer to nanotechnology. The huge surface area to volume ratio of VLNPs permits multiple copies of a targeting ligand and drugs to be attached per nanoparticle. By exploring the chemistry of truncated hepatitis B core antigen (tHBcAg) VLNPs, doxorubicin (DOX) was coupled covalently to the external surface of these nanoparticles via carboxylate groups. About 1600 DOX molecules were conjugated on each tHBcAg VLNP. Then, folic acid (FA) was conjugated to lysine residues of tHBcAg VLNPs to target the nanoparticles to cancer cells over-expressing folic acid receptor (FR). The result demonstrated that the dual bioconjugated tHBcAg VLNPs increased the accumulation and uptake of DOX in the human cervical and colorectal cancer cell lines compared with free DOX, resulting in enhanced cytotoxicity of DOX towards these cells. The fabrication of these dual bioconjugated nanoparticles is simple, and drugs can be easily conjugated with a high coupling efficacy to the VLNPs without any limitation with respect to the cargo’s size or charge, as compared with the pH-responsive system based on tHBcAg VLNPs. These dual bioconjugated nanoparticles also have the potential to be modified for other combinatorial drug deliveries.

## 1. Introduction

Over the last decade, nanomedicine has been studied intensively due to its potential applications in cancer treatments. Currently, nanoscale drug delivery systems are heavily studied to specifically target drugs into cancer cells in the interest of improving therapeutic efficacy while minimizing undesirable side effects [1,2,3,4,5,6]. As a result, various inorganic and organic nanocarriers have been developed including quantum dots, carbon nanotubes, liposomes, micelles, dendrimers, hydrogels, and biological nanocarriers. Some of these nanocarrier-based drug delivery products are in preclinical and clinical stages [7,8,9,10,11,12,13,14,15,16,17]. For instance, Doxil^®^ and Abraxane^®^ have been approved by the US Food and Drug Administration (FDA) for clinical applications [18,19]. Despite attempts to use these nanocarriers in clinical applications, their usage as a drug delivery system is limited by several issues [20]. They suffer from structural heterogeneity, particle instability, manufacturing difficulties, limited drug loading capacity, poor permeability, difficulty in preserving stability and, shelf-life, potential immunogenicity, clearance mediated by phagocytes and dendritic cells, and difficulty in controlling surface functionalization [21,22].

As an alternative, virus-like nanoparticles (VLNPs) provide an ideal basis for developing specific drug delivery systems for cancer treatments, owing to their biocompatible and biodegradable properties, monodispersity, and symmetrical structures [21,23]. One of the intensively characterised VLNPs is the hepatitis B VLNP. It is made of 180 or 240 subunits of the viral core antigen (HBcAg). A truncated HBcAg mutant (tHBcAg), lacking the arginine rich domain at its C-terminus, also self-assembles into icosahedral VLNPs [24,25,26]. tHBcAg VLNPs are very robust and stable at 70 °C for at least 1 h [27]. In addition, tHBcAg VLNPs have a large surface area, exposing a series of amino acid residues with different functional groups. As a result, different moieties can be attached and presented on the exterior surfaces of tHBcAg VLNPs. Here, we describe dual covalent modification of tHBcAg VLNPs with an anticancer drug, doxorubicin (DOX), and a tumour-targeting ligand, folic acid (FA), for specific drug delivery to the human cervical and colorectal cancer cell lines, HeLa and HT29 cells. In the present study, about 1600 DOX molecules were conjugated to tHBcAg nanoparticles via amide bonds. Then, FA molecules were conjugated to the tHBcAg VLNPs in order to specifically target the particles to the cancer cells expressing the folate receptor (FR). These dual conjugated tHBcAg VLNPs successfully delivered DOX to the HeLa and HT29 cancer cells with a higher drug payload and minimum side effects. 

In a previous study, we developed a pH-responsive drug delivery system based on tHBcAg VLNPs for controlled drug delivery [28]. Polyacrylic acid (PAA) was mixed with DOX and loaded into tHBcAg VLNPs during the reassociation of the particles. In this manner, PAA retained DOX within the tHBcAg VLNPs and prevented it from diffusing out of the VLNPs. The PAA further acted as a pH-responsive nanovalve for controlled release of DOX from the nanoparticles. Then, FA was conjugated to a pentadecapeptide containing the nanoglue bound to tHBcAg VLNPs to increase the specificity of the VLNPs. In the present study, we also compared this pH-responsive system with the newly-established dual conjugated tHBcAg VLNPs.

## 2. Results

### 2.1. Conjugation of tHBcAg VLNPs with FA

**Figure 1:**
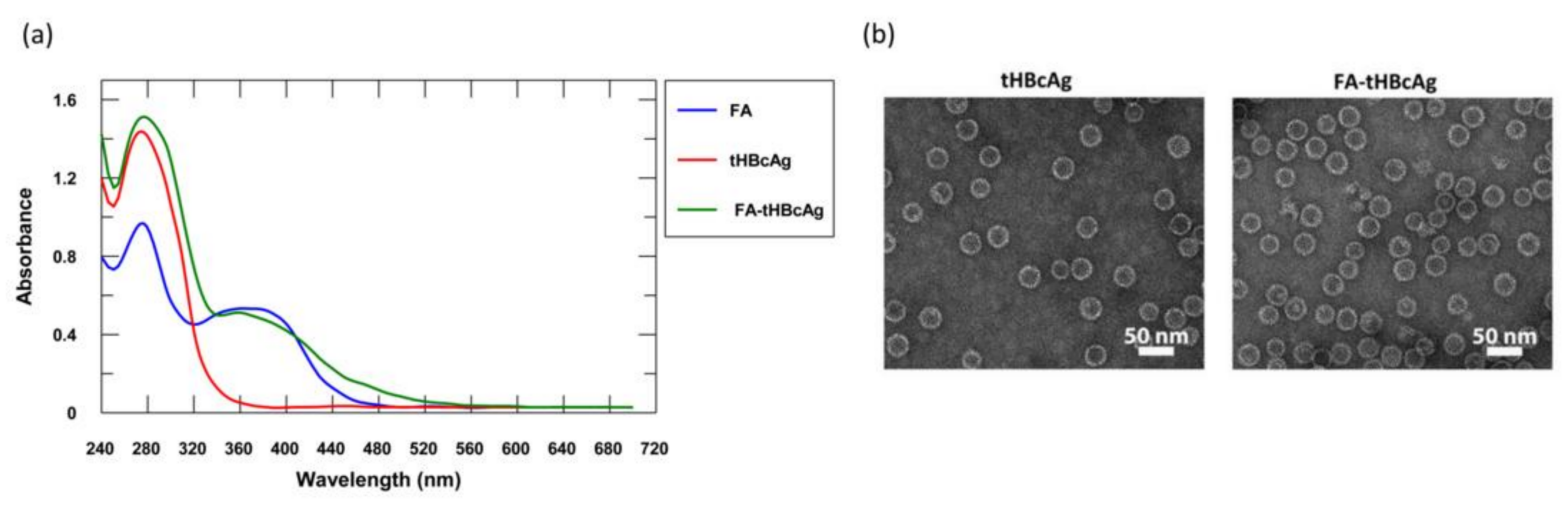
Conjugation of truncated HBcAg mutant (tHBcAg) virus-like nanoparticles (VLNPs) with folic acid. (**a**) Spectra of folic acid (FA), tHBcAg VLNPs (tHBcAg), and FA-conjugated tHBcAg VLNPs (FA-tHBcAg); (**b**) Transmission electron micrographs of tHBcAg VLNPs. tHBcAg VLNPs (tHBcAg) and FA-conjugated tHBcAg VLNPs (FA-tHBcAg) were examined using transmission electron microscopy (TEM). The white bars indicate 50 nm.

tHBcAg VLNPs were conjugated with FA via Lys residues using the 2-step carbodiimide method. The absorbance spectrum of the conjugated product, with wavelengths from 240 to 700 nm, is shown in Figure 1a. Unlike the native tHBcAg, folic acid-conjugated tHBcAg (FA-tHBcAg) exhibited a significantly higher absorbance at 360 nm (Figure 1a). A comparison with the spectrum of FA suggested that the difference in absorbance profiles is attributed to the conjugation of tHBcAg VLNPs with FA. The FA conjugation efficiency (CE_FA_) was 6.30 ± 0.40%, amounting to approximately 470 FA molecules conjugated to each VLNP or approximately 2 FA molecules conjugated to one tHBcAg subunit.

The tHBcAg VLNPs were stable throughout the FA conjugation process, and the VLNPs remained in icosahedral structure as confirmed by transmission electron microscopy (Figure 1b).

### 2.2. Internalisation of FA-Conjugated tHBcAg VLNPs into HeLa Cells

**Figure 2:**
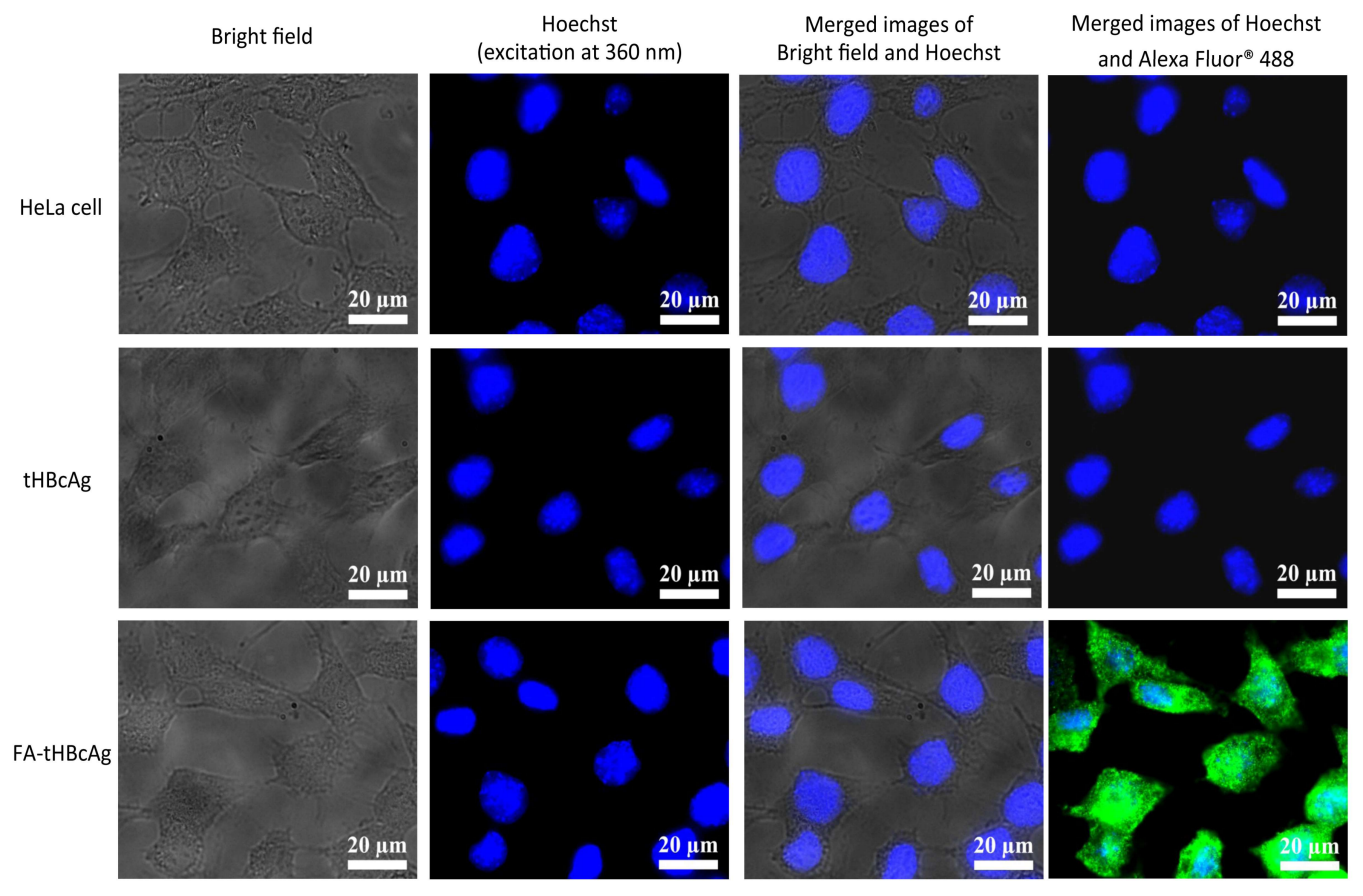
Translocation of tHBcAg VLNPs conjugated with folic acid into HeLa cells. The cancer cells were incubated with 25 μg of tHBcAg VLNPs (tHBcAg), and folic acid (FA)-conjugated tHBcAg VLNPs (FA-tHBcAg). The internalised tHBcAg VLNPs were examined using an immuno-fluorescence microscope. Non-treated HeLa cells were used as a negative control.

The internalisation property of FA-conjugated tHBcAg VLNPs into cancer HeLa cells was evaluated by immuno-fluorescence microscopy. The rabbit anti-tHBcAg antibody and the anti-rabbit Immunoglobulin (IgG) conjugated to Alexa Fluor 488 were used to detect internalised tHBcAg VLNPs. The results showed that the FA-conjugated tHBcAg VLNPs internalised into HeLa cells and emitted green fluorescence (Figure 2).

HeLa cells treated with FA-tHBcAg VLNPs showed an intense green fluorescent signal in the cytoplasm (Figure 2). The accumulation of FA-tHBcAg VLNPs in the cytoplasm of the cells indicates the FR-mediated internalisation of these VLNPs into the HeLa cells. In this experiment, tHBcAg VLNPs were used as a negative control. These VLNPs did not internalise HeLa cells as they did not produce any green fluorescent signal. Also, the fluorescent signal was not detected in the untreated HeLa cells (Figure 2).

### 2.3. Conjugation of Doxorubicin and Folic Acid to tHBcAg VLNPs

**Figure 3:**
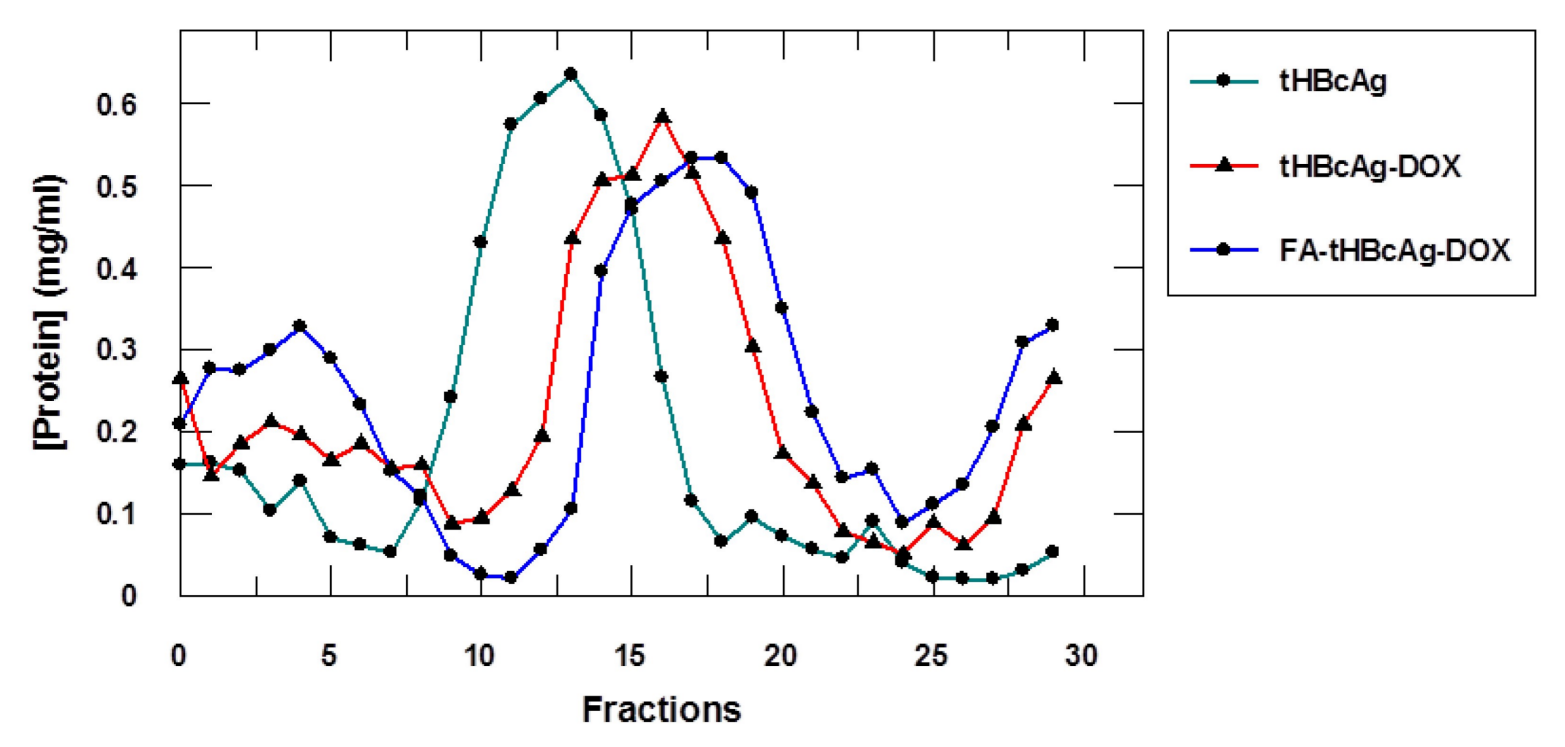
Migration profile of the tHBcAg VLNPs conjugated with doxorubicin (DOX) and folic acid (FA) in the sucrose gradient. tHBcAg VLNPs (tHBcAg), tHBcAg VLNPs conjugated with DOX (tHBcAg-DOX), and tHBcAg VLNPs conjugated with DOX and FA (FA-tHBcA-DOX) were separated on sucrose gradients, and the total amount of protein in each fraction was measured using the Bradford assay. tHBcAg served as a negative control.

The surface-exposed carboxylate groups of tHBcAg VLNPs were conjugated with DOX using the EDC/Sulfo-NHS method and studied with sucrose density gradient ultracentrifugation. tHBcAg VLNPs conjugated with DOX (tHBcAg-DOX) migrated in the sucrose gradient and showed a peak in fractions 11–23, whereas non-conjugated VLNPs stayed in fractions 8–18 (Figure 3). The faster migration of tHBcAg-DOX VLNPs in the sucrose gradient was due to the presence of denser particles, indicating that DOX was conjugated to the tHBcAg VLNPs.

The tHBcAg-DOX VLNPs were then conjugated with FA, to produce FA-tHBcAg-DOX VLNPs. The result obtained from sucrose density gradient ultracentrifugation indicated that FA-tHBcAg-DOX VLNPs migrated into the gradient and accumulated in fractions 13–25 (Figure 3). The sedimentation rate of the FA-tHBcAg-DOX VLNPs was higher compared with that of the tHBcAg-DOX VLNPs, demonstrating that FA was successfully conjugated to the tHBcAg-DOX VLNPs.

**Figure 4:**
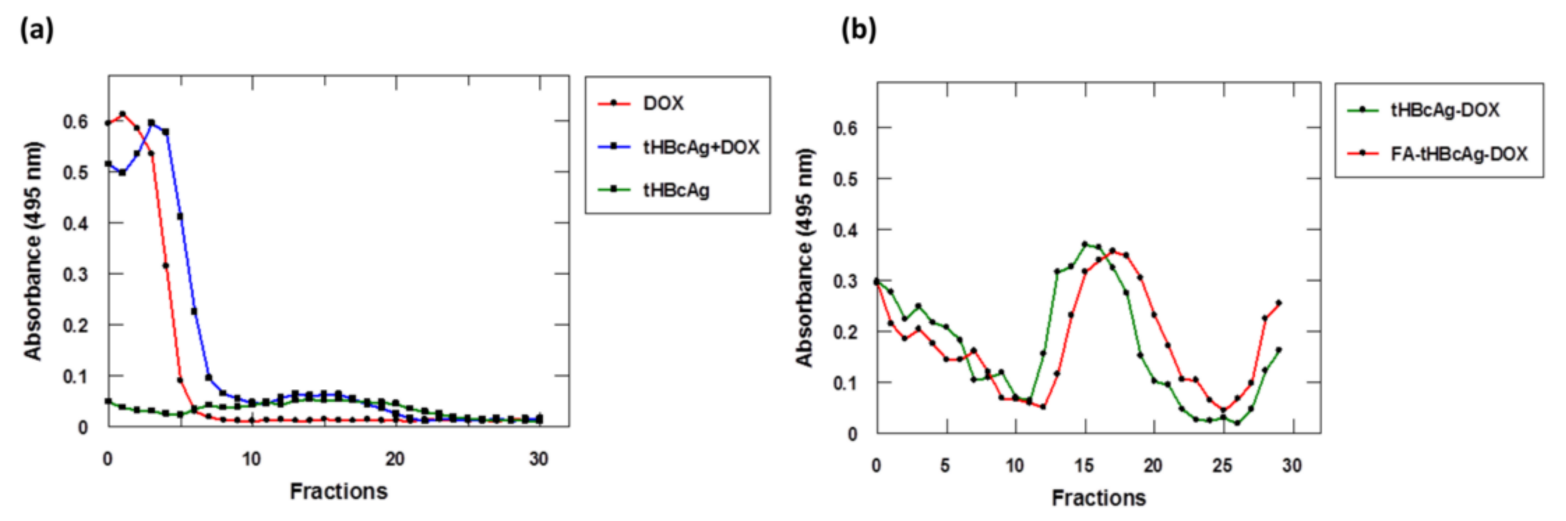
Detection of doxorubicin conjugated to tHBcAg VLNPs separated on sucrose gradients. The absorbance at 495 nm (A_495_) of samples was measured with a spectrophotometer. (**a**) Doxorubicin (DOX), and the DOX added to tHBcAg without the conjugation procedure (tHBcAg + DOX) stayed on top of the sucrose gradients. tHBcAg VLNPs (tHBcAg), which served as a negative control, were not detected at A_495_; (**b**) DOX-conjugated tHBcAg VLNPs (tHBcAg-DOX), as well as tHBcAg VLNPs conjugated with folic acid (FA) and DOX (FA-tHBcAg-DOX) migrated into the sucrose gradients, and the conjugated DOX was detected at A_495_.

To detect the DOX conjugated to tHBcAg VLNPs, the absorbance at 495 nm (A_495_) of samples was measured using a spectrophotometer. The results showed that the DOX alone, and DOX from tHBcAg + DOX (DOX added to tHBcAg without conjugation) stayed on top of the sucrose gradients (Figure 4a). The tHBcAg VLNPs did not exhibit any noticeable absorbance at A_495_. In contrast, DOX-conjugated samples were detected in fractions 12–22 and 14–25, of the tHBcAg-DOX and FA-tHBcAg-DOX VLNPs, respectively (Figure 4b). This suggests that the conjugation of DOX and FA to the tHBcAg VLNPs increased the density of the VLNPs, resulting in shifted migration towards denser fractions.

**Figure 5:**
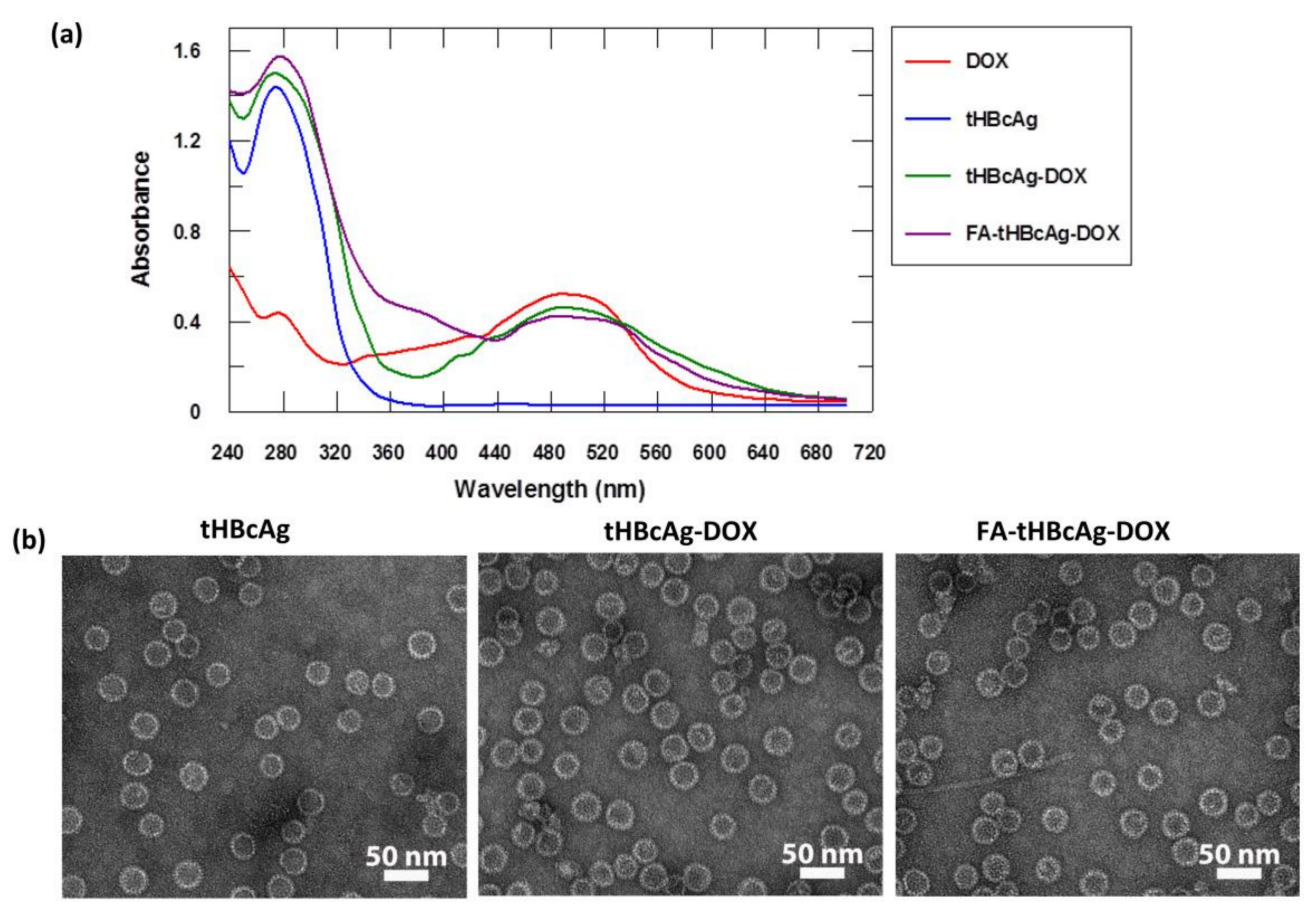
Conjugation of tHBcAg VLNPs with doxorubicin and folic acid. (**a**) Spectra of doxorubicin (DOX), tHBcAg VLNPs (tHBcAg), tHBcAg VLNPs conjugated with DOX (tHBcAg-DOX), and tHBcAg VLNPs conjugated with folic acid (FA) and DOX (FA-tHBcAg-DOX); (**b**) Transmission electron micrographs of tHBcAg VLNPs conjugated with DOX and FA. Nanoparticles formed by tHBcAg VLNPs (tHBcAg), tHBcAg VLNPs conjugated with DOX (tHBcAg-DOX), and tHBcAg VLNPs conjugated with DOX and FA (FA-tHBcAg-DOX). The white bars indicate 50 nm.

The sucrose fractions containing tHBcAg-DOX and FA-tHBcAg-DOX VLNPs were pooled and characterised with a spectrophotometer from wavelengths 240 nm to 700 nm (Figure 5a). tHBcAg-DOX VLNPs exhibited two absorbance peaks at 280 nm and 495 nm. The former peak indicates the presence of tHBcAg VLNPs, and the latter peak indicates that DOX was conjugated to the nanoparticles. For FA-tHBcAg-DOX VLNPs, three peaks were observed at A_280_, A_360_ and A_495_. The second peak indicates that FA was successfully conjugated to tHBcAg VLNPs, while the third peak indicates that the nanoparticles were conjugated with DOX (Figure 5a).

Approximately 1600 DOX molecules were conjugated to each FA-tHBcAg VLNP with a conjugation efficiency (CE_DOX_) of 21.75 ± 0.80%. Transmission electron micrographs revealed that tHBcAg VLNPs were stable during the conjugation process and formed spherical nanoparticles (Figure 5b). The structures of tHBcAg-DOX and FA-tHBcAg-DOX VLNPs were similar to that of tHBcAg VLNPs.

### 2.4. Migration Profile of tHBcAg VLNPs Conjugated with Doxorubicin and Folic Acid in a Native Agarose Gel 

**Figure 6:**
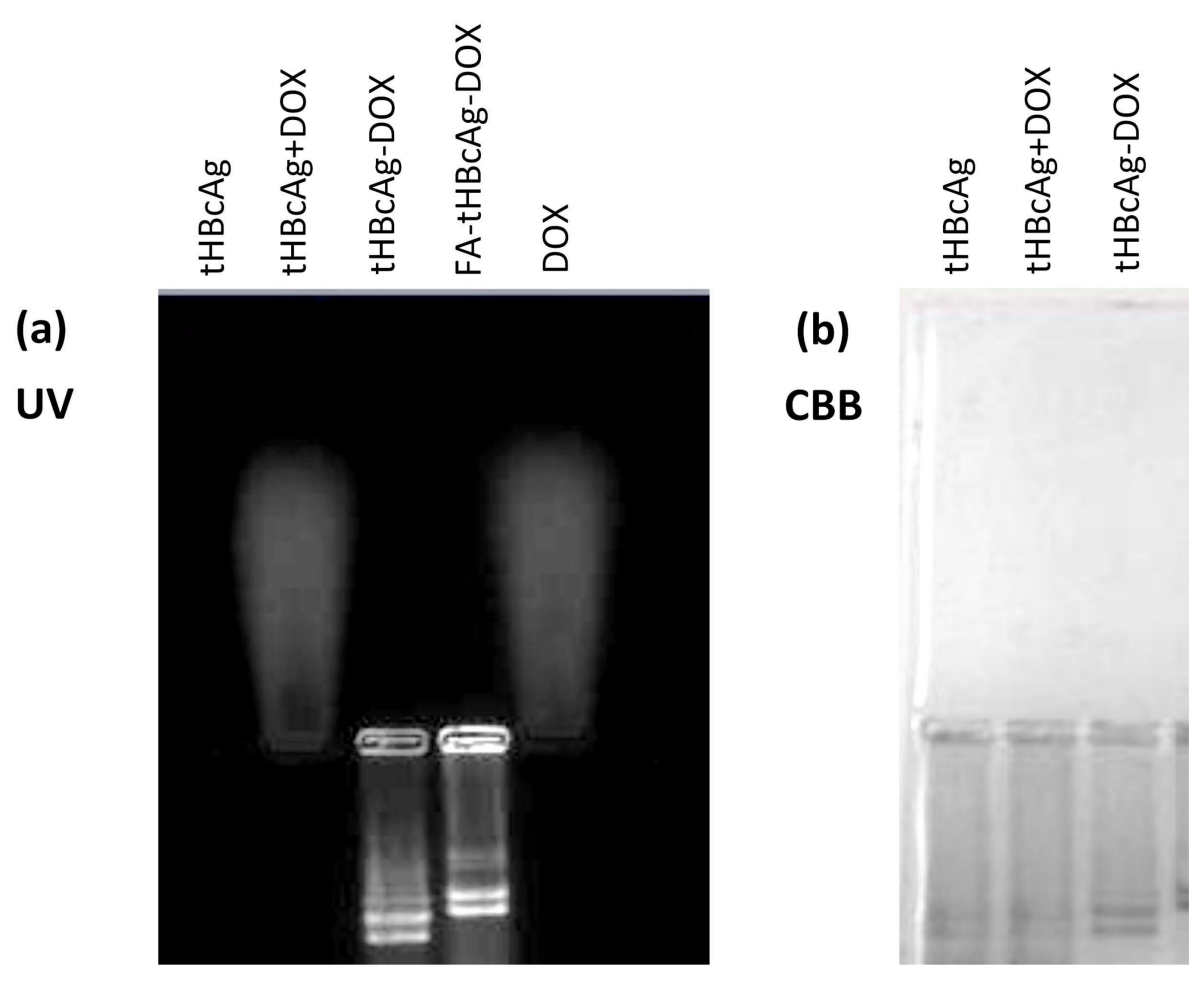
Migration profile of the tHBcAg VLNPs conjugated with folic acid and doxorubicin in a native agarose gel. The same gel was (**a**) visualised under ultraviolet (UV) illumination and (**b**) stained with Coomassie brilliant blue (CBB). Samples labelled on top of the gel images are tHBcAg VLNPs (tHBcAg), tHBcAg VLNPs incubated with doxorubicin (DOX) without the conjugation steps (tHBcAg + DOX), tHBcAg VLNPs conjugated with DOX (tHBcAg-DOX), tHBcAg VLNPs conjugated with folic acid (FA) and DOX (FA-tHBcAg-DOX), and free DOX.

Figure 6 shows the migration profile of DOX-conjugated tHBcAg VLNPs and free DOX in an agarose gel. Free DOX and DOX-conjugated tHBcAg VLNPs could be observed in the native agarose gel under UV light because of the inherent fluorescence of the DOX. The gel exposed to UV light showed that free DOX migrated towards the negative electrode (cathode) and fluoresced, while tHBcAg VLNPs did not fluoresce (Figure 6a). When DOX was conjugated to tHBcAg VLNPs, the tHBcAg-DOX and FA-tHBcAg-DOX VLNPs migrated towards the positive electrode (anode) and fluoresced, indicating co-migration of DOX with tHBcAg VLNPs (Figure 6a). When proteins were stained with Coomassie brilliant blue (CBB), the conjugated and native tHBcAg VLNPs were observed. The native tHBcAg VLNPs migrated to the anode (Figure 6b). Conjugation of DOX and FA to tHBcAg VLNPs slowed down the migration profiles of the carriers (Figure 6b). These migration profiles were consistent with those of the gel exposed to UV light. Co-incubation of tHBcAg VLNPs and DOX without the conjugation steps (tHBcAg + DOX) resulted in migration of DOX and tHBcAg nanoparticles to the cathode and anode, respectively. This demonstrates that DOX did not attach to the surfaces of tHBcAg VLNPs (Figure 6).

### 2.5. DOX Uptake by Cancer and Normal Cells

**Figure 7:**
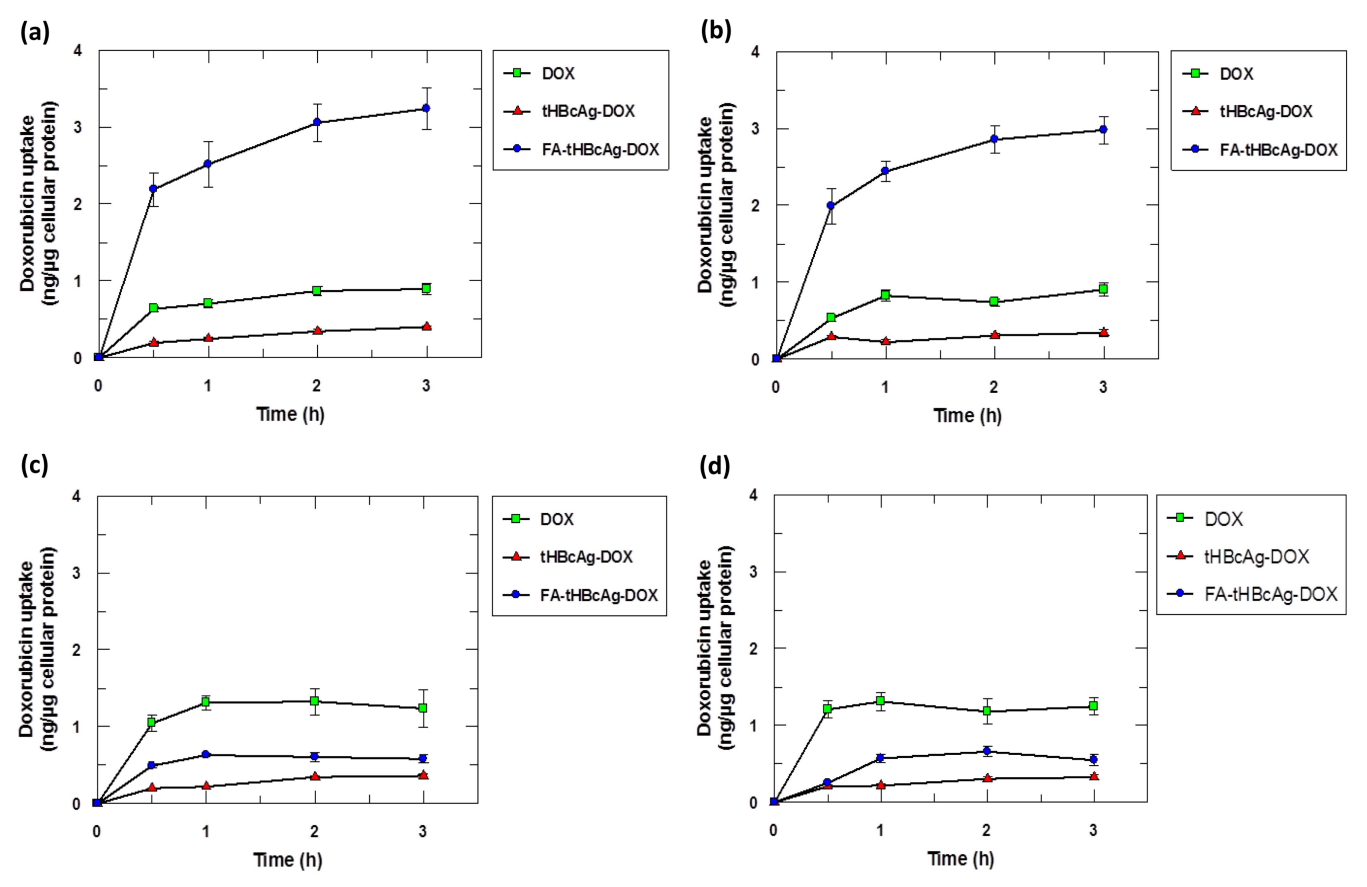
Doxorubicin (DOX) uptake by cancer and normal cells. DOX uptake by cancer (**a**) HeLa and (**b**) HT29 cells, and normal (**c**) 3T3 and (**d**) CCD-112 cells incubated with DOX alone, DOX-conjugated tHBcAg VLNPs (tHBcAg-DOX), dual-conjugated tHBcAg VLNPs with folic acid (FA) and DOX (FA-tHBcAg-DOX). Data represent means ± standard deviations (SD) of triplicate determinations.

DOX uptake by cancer and normal cells from free DOX, tHBcAg-DOX VLNPs, and FA-tHBcAg-DOX VLNPs was quantified spectrophotometrically and monitored using live cell imaging microscopy. The quantitative data revealed a significantly higher cellular uptake of DOX from FA-tHBcAg-DOX VLNPs by HeLa and HT29 cells compared to free DOX and tHBcAg-DOX VLNPs (Figure 7a,b). This indicates a FR-mediated uptake mechanism by the HeLa and HT29 cells. By contrast, the cellular uptake of FA-tHBcAg-DOX VLNPs by normal 3T3 and CCD-112 cells was significantly lower compared to free DOX (Figure 7c,d).

**Figure 8:**
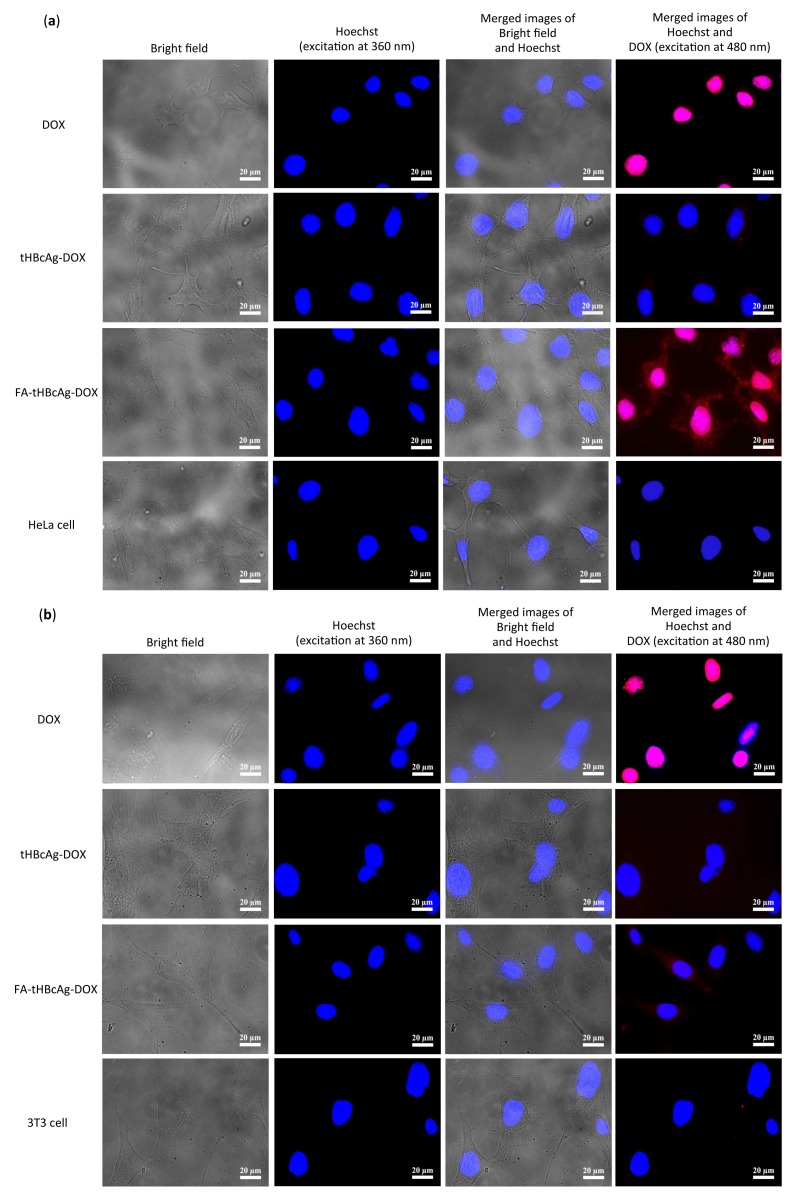

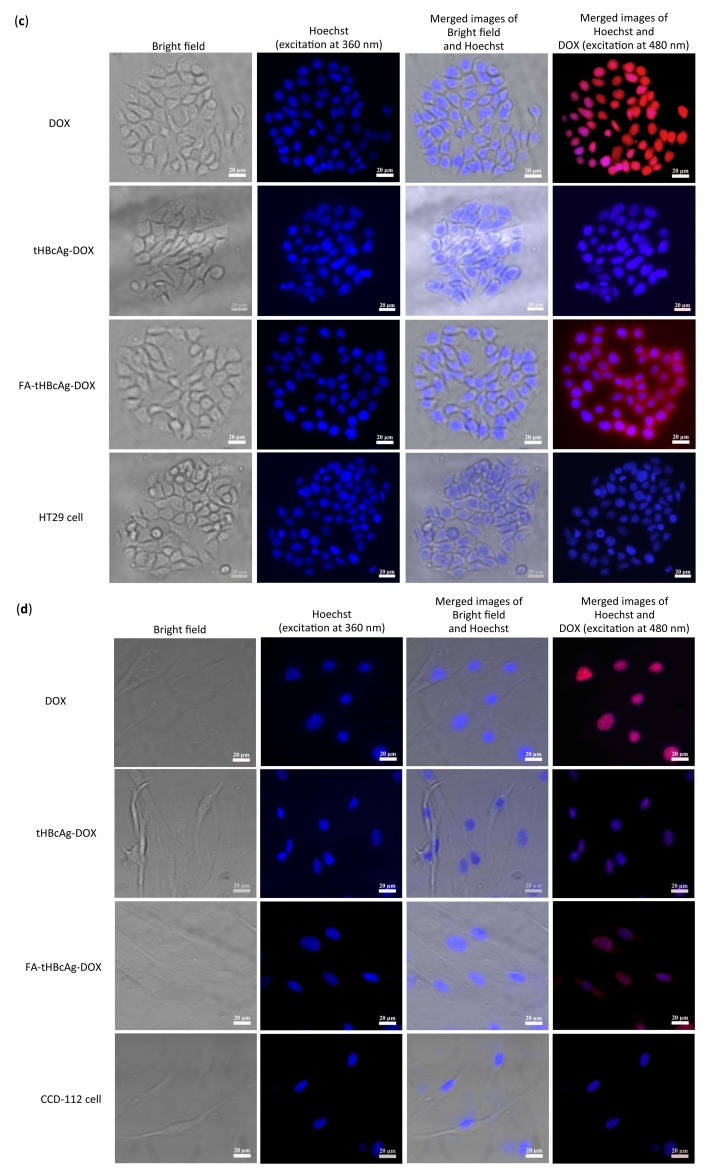
Delivery of doxorubicin by dual conjugated tHBcAg VLNPs into cancer (HeLa, HT29) and normal (3T3, CCD-112) cells. (**a**) Human cervical cancer HeLa; (**b**) normal 3T3; (**c**) human colorectal cancer HT29; and (**d**) normal CCD-112 cells were treated with equivalent DOX concentration (5 μg/mL) of doxorubicin (DOX) alone, DOX-conjugated tHBcAg VLNPs (tHBcAg-DOX), and tHBcAg VLNPs conjugated with folic acid (FA) and DOX (FA-tHBcAg-DOX) for 1 h. The cells without treatment served as negative controls. DOX was detected at 480 nm. Scale bars indicate 20 μm.

Live cell imaging micrographs also provided supporting evidence for the FR-mediated preferential uptake of FA-tHBcAg-DOX VLNPs by HeLa and HT29 cells. Figure 8a,c show that FA-tHBcAg-DOX VLNPs internalised the HeLa and HT29 cells, respectively. Intense red fluorescence was observed in the cytoplasm of these cells, indicating the presence of DOX in these compartments (Figure 8a,c). By contrast, the normal 3T3 and CCD-112 cells incubated with tHBcAg-DOX and FA-tHBcAg-DOX VLNPs did not exhibit intense red fluorescence. The fluorescent intensity of free DOX in these cells was higher than that of tHBcAg-DOX and FA-tHBcAg-DOX VLNPs (Figure 8b,d).

### 2.6. Cytotoxicity of tHBcAg VLNPs Conjugated with Doxorubicin and Folic Acid 

**Figure 9:**
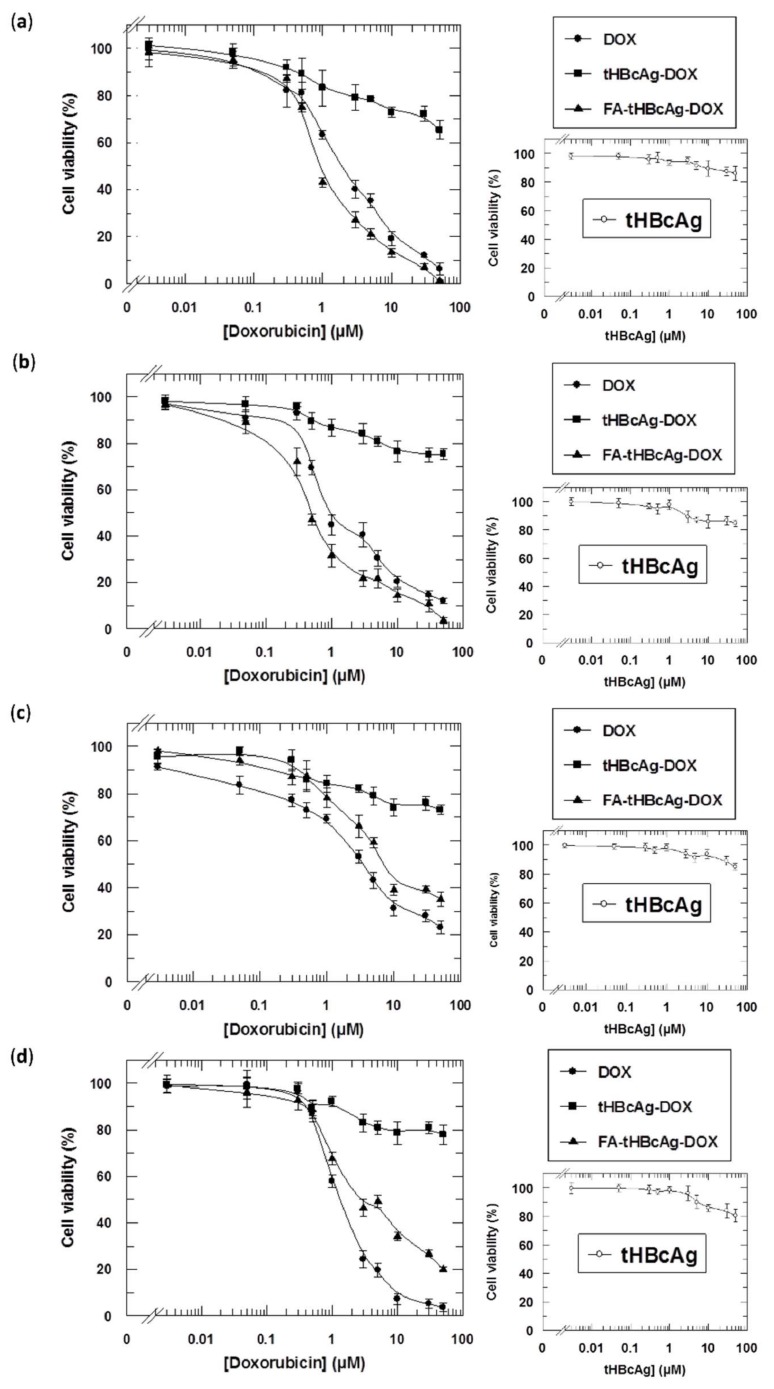
Viability of cancer and normal cells after being treated with tHBcAg VLNPs conjugated with doxorubicin and folic acid at equal DOX concentrations. Viability of cancer (**a**) HeLa; (**b**) HT29 cells; and normal (**c**) 3T3; (**d**) CCD-112 cells. tHBcAg VLNPs conjugated with folic acid (FA) and doxorubicin (DOX) (FA-tHBcAg-DOX) were more efficient in inhibiting the growth of cancer HeLa and HT29 cells than other formulations. By contrast, conjugation of DOX to the FA-conjugated tHBcAg VLNPs led to a decrease in cytotoxicity on normal 3T3 and CCD-112 cells, resulting in protection of the normal cells against DOX. Data represent means ± SDs of triplicate determinations. The small graphs on the right show that tHBcAg VLNPs (tHBcAg) are not toxic to the tested cancer and normal cells.

To evaluate the toxicity of the dual conjugation of DOX and FA to tHBcAg VLNPs, an MTT [3-(4,5-dimethylthiazol-2-yl)-2,5-diphenyltetrazolium bromide] assay was carried out by incubating cancer (HeLa, HT29), and normal (3T3, CCD-112) cells with different concentrations of FA-tHBcAg-DOX VLNPs, tHBcAg-DOX VLNPs, and free DOX. FA-tHBcAg-DOX VLNPs showed a higher toxicity against both cancer HeLa and HT29 cells. The IC_50DOX_ of FA-tHBcAg-DOX VLNPs against HeLa cells was 0.93 ± 0.09 μM, while the IC_50DOX_ of free DOX was 1.98 ± 0.10 μM (Figure 9a). The IC_50DOX_ of FA-tHBcAg-DOX VLNPs and free DOX against HT29 cells was 0.44 ± 0.04 μM and 0.90 ± 0.08 μM, respectively (Figure 9b). The IC_50DOX_ of FA-tHBcAg-DOX VLNPs and free DOX against normal 3T3 cells was 6.82 ± 0.39 μM and 3.10 ± 0.29 μM, respectively (Figure 9c). The IC_50DOX_ of FA-tHBcAg-DOX VLNPs and free DOX against CCD-112 cells was 3.01 ± 0.25 μM and 1.22 ± 0.11 μM, respectively (Figure 9d).

## 3. Discussion

**Scheme 1:**
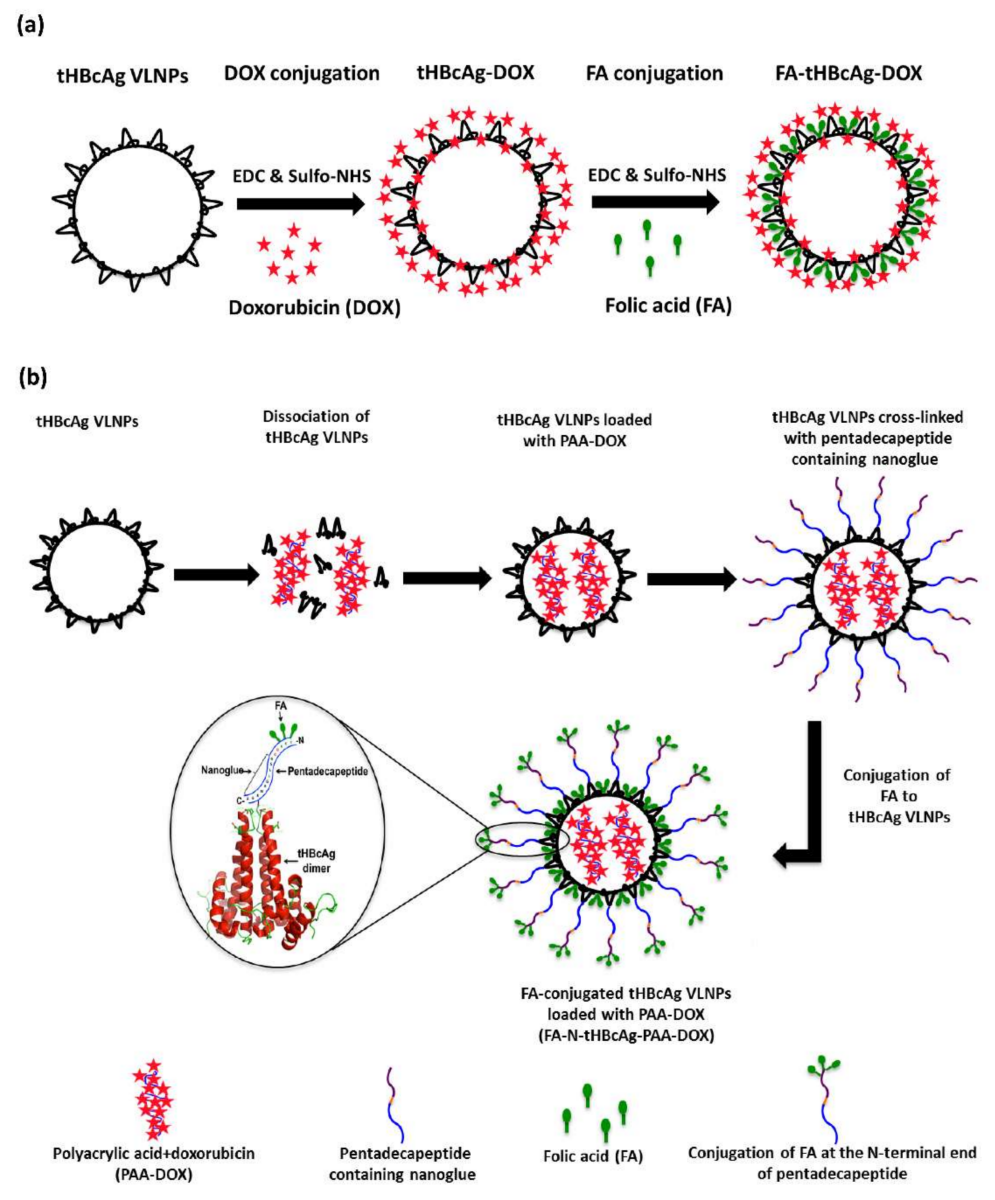
Schematic representation of two different drug delivery systems based on VLNPs. (**a**) Dual conjugated tHBcAg VLNPs. tHBcAg VLNPs possess several accessible Glu, Asp and Lys residues for the conjugation of drug and cancer targeting ligands. The carboxylate groups of Glu and Asp are used to conjugate doxorubicin (DOX). DOX-conjugated VLNPs are then conjugated with folic acid (FA) at Lys residues; (**b**) pH-responsive tHBcAg VLNPs. tHBcAg VLNPs are dissociated into tHBcAg dimers using urea. DOX is mixed with polyacrylic acid (PAA) and added into the mixture containing dissociated tHBcAg dimers. When the urea is removed by dialysis, tHBcAg dimers reassemble into nanoparticles and package PAA-DOX. tHBcAg VLNPs loaded with PAA-DOX are then cross-linked with a pentadecapeptide containing the nanoglue. Cross-linked VLNPs are then conjugated with FA via the three Lys residues located at the N-terminal end of the pentadecapeptide using EDC [1-ethyl-3-(3-dimethyl-aminopropyl)-carbodiimide hydrochloride] and Sulfo-NHS (N-hydroxysulfosuccinimide).

In this study, a dual conjugated drug delivery system was developed by taking advantage of the reactive surface groups of tHBcAg VLNPs (Scheme 1a). Approximately 1600 DOX and 470 FA molecules were successfully conjugated to the external surface of each tHBcAg VLNP via carboxylate and primary amine groups, respectively, using the EDC/Sulfo-NHS method. The TEM analysis showed that the tHBcAg VLNPs preserved their icosahedral structure when DOX and FA were conjugated to them.

Live cell imaging revealed that the cellular uptake of dual conjugated FA-tHBcAg-DOX VLNPs by the cancer cells (HeLa and HT29) was much higher compared with free DOX. This indicates that the uptake of these nanoparticles by the cancer cells was via FR-mediated endocytosis. On the other hand, the cellular uptake of these dual conjugated VLNPs by the normal cells (3T3 and CCD-112) was much lower compared with the cancer cells. This can be explained by the fact that the FR is highly expressed in HeLa and HT29 cancer cells [29,30], but it is lowly expressed in normal cells [28,31]. In addition, cancer cells overexpress the α-FR which has a high affinity to the free R-carboxylic acid of the conjugated FA molecules, whereas, normal cells mostly express the β-FR which has a higher binding affinity for 5-methyltetrahydrofolate, the reduced form of FA [32]. This is in line with our finding in which FA-tHBcAg-DOX VLNPs were taken up at much higher rates by the cancer cells (HeLa and HT29) than the normal cells (CCD-112 and 3T3).

The dual conjugated FA-tHBcAg-DOX VLNPs were more cytotoxic to cancer HeLa and HT29 cells compared with free DOX. The IC_50DOX_ of FA-tHBcAg-DOX VLNPs was about two-fold lower compared with that of free DOX. This decrement could be explained by the uptake mechanism of FA-tHBcAg-DOX VLNPs and free DOX. Free DOX molecules diffuse through HeLa and HT29 cell membranes and target the nucleus, while FA-tHBcAg-DOX VLNPs can enter the cells via FR-mediated endocytosis by binding to FR which is highly expressed in HeLa and HT29 cells. Therefore, FA-tHBcAg-DOX VLNPs enhance the cellular uptake of DOX and increase its efficiency at low drug concentrations. By contrast, both the normal 3T3 and CCD-112 cells incubated with FA-tHBcAg-DOX VLNPs showed a conferred protection against DOX, as the IC_50DOX_ of this formulation increased by approximately 2.20 and 2.47-fold, respectively, compared with that of free DOX. Bredehorst et al. [33] demonstrated that HBcAg VLNPs were stable in serum for 10 days at room temperature without any degradation, but Yap et al. [34] reported that these nanoparticles are highly immunogenic and elicit strong B-cell and T-cell immune responses. The immunogenicity of HBcAg VLNPs can be reduced by deleting the major immunodominant region (residues 79–81) of the HBcAg monomer [35]. These mutated HBcAg VLNPs can be conjugated with DOX and FA, and their immunogenicity and stability in the bloodstream can be studied in an animal model.

In a previous study, we established a pH-responsive drug delivery system. FA was conjugated to a pentadecapeptide containing nanoglue bound to tHBcAg VLNPs, and DOX was loaded into the VLNPs during the reassociation of the particles (Scheme 1b). Table 1 summarizes the differences between the pH-responsive and dual conjugated drug delivery systems. In the former system, conjugation of FA onto tHBcAg VLNPs using the nanoglue (FA-N-tHBcAg-PAA-DOX) significantly enhanced the number of FA molecules conjugated to the VLNPs and the cellular uptake and accumulation of DOX in cancer cells, leading to enhanced anti-tumour effects. The expression of FR has been shown to increase considerably with the stage of cancer [36], thus this system could be used as a potential therapy for advanced cancers. 

For the dual conjugated drug delivery system, the number of DOX molecules conjugated to tHBcAg VLNPs was significantly higher compared to DOX loaded in the pH-responsive tHBcAg VLNPs. Approximately 1600 DOX molecules were conjugated to each FA-tHBcAg VLNP with a conjugation efficiency (CE_DOX_) of 21.75 ± 0.80%, while 946 DOX molecules were packaged in each FA-N-tHBcAg VLNP with a loading efficiency (LE_DOX_) of 12.90 ± 0.50%. Technically, conjugation is simpler and can be performed more easily with a high coupling efficacy. However, the cytotoxicity of the dual conjugated VLNPs was lower than that of the pH-responsive VLNPs. This could be due to the fact that FA-tHBcAg-DOX VLNPs enter the cancer cells via FR-mediated endocytosis, and the drug can be released when the particles enter endosomes and are degraded. Therefore, these dual conjugated VLNPs are not pH-responsive. On the other hand, the pH-responsive VLNPs enter the cancer cells via FR-mediated endocytosis, and the encapsulated drug is released at tumour sites in a controlled manner, resulting in a higher therapeutic efficiency. 

In the dual conjugated drug delivery system, molecules of interest, such as drugs or tumour targeting ligands can be coupled covalently to amine or carboxyl groups of nanoparticles. This method increases the stability of the nanoparticles compared to the passive absorption approach. Covalent conjugation employs chemical linkers which react specifically with certain chemical groups exposed on the surface of nanoparticles. Thus, the conjugation approach is more specific and controllable than the passive absorption approach. To date, conjugation of anti-cancer drugs such as DOX to nanocarriers is one of the most common approaches for developing drug delivery systems [37,38]. Conjugation of anti-cancer drugs such as DOX on the surface of nanoparticles enhances the therapeutic efficacy and biological specificity of the drugs and minimizes systemic toxicity [37,39,40]. The covalent conjugation of drugs is mainly used for targeting and achieving a higher drug payload [41,42]. Cross-linkers are commercially available for the cross-linking of drugs and ligands with different sizes and charges to VLNPs. Covalent coupling through a linker could minimize steric hindrance and impact on the tertiary structures of conjugated VLNPs, resulting in less deleterious effects on the properties of the nanoparticles. However, chemical conjugation may have limited control over the number of VLNP modifications, leading to heterogeneous and incomplete VLNPs conjugation with drugs [43]. In addition, conjugation using EDC/Sulfo-NHS may cross-link two or more VLNPs together if these particles have carboxylate and primary amine groups on their surfaces. In the present study, tHBcAg VLNPs possess a series of carboxylate and amine groups which can be cross-linked to each other during the conjugation. To overcome this issue, the carboxylate groups of tHBcAg VLNPs were first activated by Sulfo-NHS (N-hydroxysulfosuccinimide) and EDC [1-ethyl-3-(3-dimethyl-aminopropyl)-carbodiimide hydrochloride] at pH 6. Then the pH of solution was raised to 7.4 to cross-link DOX to tHBcAg VLNPs. This resulted in a higher conjugation efficiency between DOX and VLNPs and prevented cross-linking among the VLNPs. In addition, the conjugation of drugs to VLNPs may affect the solubility of conjugated products, thus it is important to study their zeta potentials which provide insight into the causes of aggregation and dispersion.

Loading drugs into VLNPs improves the solubility of insoluble drugs [41,42,44] and protects labile drugs from degradation in biological environments [44,45]. However, a drug must possess a certain charge and size in order to be packaged in a VLNP. In this study, DOX did not fulfill the criteria to be packaged inside tHBcAg VLNPs. Therefore, DOX was mixed with PAA and packaged inside the VLNPs during the reassociation of the nanoparticles. The pKas of PAA and DOX are 4.8 and 8.6, respectively [28]; thus an electrostatic interaction takes place between negatively charged PAA and positively charged DOX at pH 7.4, and this interaction is reversible at low pH. At the physiological pH of normal tissues, DOX is retained in tHBcAg nanoparticles and it is only released when the nanoparticles reach extracellular tissues of tumours or intracellular endosomes with a pH of around 5–5.5. Consequently, DOX can be loaded inside the VLNPs without any modification, and the pharmacological activity of the loaded DOX will be preserved. The solubility of the drug increases, and it is protected from enzymatic degradation in the human body.

In the present study, the dual conjugated tHBcAg VLNPs successfully delivered DOX to HeLa and HT29 cancer cells with a higher drug payload and lowered the drug’s cytotoxicity. To date, different organic and inorganic nanoparticles have been employed for cancer therapy which specifically deliver chemotherapeutics to cancer cells [8,17,46,47,48]. Although these nanoparticles offer several advantages as drug delivery systems, they have some drawbacks [16]. Liposomal nanoparticles suffer from particle instability, spontaneous membrane fusion to non-target cells, and rapid clearance in the bloodstream [49,50]. Polymer-based nanoparticles are limited by structural heterogeneity, slow drug release, particle instability, and potential immunogenicity when the particles are tested in vivo [21,51,52]. The metal-based nanoparticles with a higher stability are still limited by high toxicity and lack of specificity [53]. In addition, most of these nanoparticles suffer from phagocytic clearance by Kupffer and dendritic cells in the liver. Although the coating of nanoparticles with polyethylene glycol (PEG) could extend the blood circulation time and avoid phagocytes [54,55], PEGylation could reduce the uptake of nanoparticles by the targeted cells because they are potentially immunogenic [21,51,52]. In addition, surface functionalization of these nanoparticles is difficult to control, which results in non-uniform particles [22]. Layer-by-layer (LbL) films have been introduced to address most of these challenges [56]. By using LbL techniques, various parameters can be precisely controlled, such as chemistry, thickness, and mechanical properties, which improve the stability of LbL carriers [57,58]. In addition, LbL-assembled nanoparticles can control the quantity of anti-cancer drug entrapped inside the particles, and its subsequent release at targeted sites [57,59]. Despite the many advantages of LbL nanoparticles, many challenges remain to be addressed, including the following: (i) The size of LbL nanoparticles needs to be improved in order to escape phagocytosis in the bloodstream and accumulate in tumour cells through the enhanced permeability and retention (EPR) effect; (ii) the mechanical properties need to be improved to allow a longer period of circulation in the bloodstream; (iii) the downstream cellular responses and signaling pathways during the interactions of LbL-based nanoparticles with targeted cells or organs require more investigation [57]; (iv) the ability to form coatings in a time- and cost-effective manner; and (v) the ability to control interlayer diffusion [56].

As an alternative, VLNPs have received attention as a smart drug delivery system which could overcome the limitations of other nanoparticles [21]. VLNPs offer many advantages over synthetic nanomaterials: (i) VLNPs have highly-ordered architecture with structural stability on the nanosize scale, which is essential for tumour permeability and retention [60]; (ii) the homogeneity in size and shape as well as the ease of production compared to those of other drug delivery nanoparticles, such as lipids and polymers which are prepared through random aggregation [61]; (iii) biocompatibility and biodegradability; (iv) three distinct interfaces (external, internal and inter-subunits) are available for functionalization; (v) both genetic and chemical modifications may be used; and (vi) repetitive structure which means one modification aligns the whole particle in a constant manner [62].

VLNPs are much less toxic during parenteral administration than metal nanoparticles, more stable than liposomes, and more uniform than polymer nanoparticles [21,63,64]. However, VLNPs do have drawbacks. Like other nanoparticles, the major challenge of VLNPs is phagocytic clearance, even with PEGylated VLNPs [65]. In addition, recent research has shown that ellipsoid nanoparticles are able to extravasate more effectively from the blood vessel than spherical nanoparticles [66]. Polymeric nanoparticles can be fabricated to adopt an ellipsoid shape, but this is not feasible for icosahedral VLNPs. However, by displaying multiple ligands with high affinity for the tight junctions between endothelial cells, VLNPs may be able to efficiently extravasate from the vasculature of the blood vessels [21].

## 4. Materials and Methods

### 4.1. Expression and Purification of tHBcAg VLNPs

The *E. coli* strain, W3110IQ, carrying pR1–11E plasmid was used to produce tHBcAg (residues 3–148), as described by Tan et al. [25]. The tHBcAg VLNPs were purified by a high-performance liquid chromatography (HPLC) system (Agilent 1100 Series, Agilent, Santa Clara, CA, USA) as described by Tang et al. [67] with some modifications. The tHBcAg in bacteria lysate was purified with a Zorbax Bio Series GF-450 column (Agilent, Santa Clara, CA, USA), using TBS buffer (50 mM Tris-HCl, 100 mM NaCl, pH 8.0) at a flow rate of 1.0 mL/min. The purity of the tHBcAg was analysed with SDS-PAGE, and the protein concentration was determined using the Bradford assay [68]. 

### 4.2. Conjugation of tHBcAg VLNPs with Folic Acid 

The carboxylic acid groups of FA were activated by Sulfo-NHS and EDC, according to the method described by Biabanikhankahdani et al. [32]. Then, the activated FA molecules were added to the solution of tHBcAg VLNPs in sodium phosphate buffer (100 mM Na_2_HPO_4_/NaH_2_PO_4_, pH 7.4), and the FA conjugated VLNPs were separated by sucrose density gradient (8%–40%, *w*/*v*) as explained by Biabanikhankahdani et al. [28]. The fractions containing the highest amount of FA-conjugated nanoparticles were collected and dialysed against sodium phosphate buffer (1 L, 4 °C) using 12 kDa cut-off membranes (Sigma-Aldrich, St. Louis, MO, USA) and concentrated with VIVASPIN 20 (30 kDa cut-off, Sigma-Aldrich, St. Louis, MO, USA). 

### 4.3. UV-Visible Spectroscopy

Absorbance at 360 nm (A_360_) of FA-conjugated tHBcAg VLNPs was measured using a NanoDrop^TM^ 1000 spectrophotometer (Thermo Scientific, Rockford, IL, USA) at room temperature. The conjugated FA was quantified using an extinction coefficient of 5312 mol^−1^ cm^−1^, as described by Ren et al. [69]. The conjugation efficiency of FA (CE_FA_) and the number of FA (N_FA_) molecules conjugated to each nanoparticle were calculated using Equations (1) and (2), respectively. 

CE_FA_% = weight_FA_/weight_tHBcAg particle_ × 100% (1)

N_FA_ = CE_FA_ × (Mw_tHBcAg particle_/Mw_FA_) (2)

### 4.4. Cancer and Normal Cell Lines

The human cervical cancer cell line (HeLa), colorectal cancer cell line (HT29), and normal cell lines (3T3 and CCD-112) were obtained from the American Type Culture Collection (ATCC). HeLa and HT29 cell lines were grown in FA-deficient GIBCO RPMI1640 medium (Life Technology, Grand Island, NY, USA) while the 3T3 and CCD-112 cells were cultured continuously in DMEM and EMEM media, respectively (Sigma, St. Louis, MO, USA), containing heat-inactivated fetal bovine serum (FBS, 10%; Sigma, St. Louis, MO, USA) as a monolayer. The cells were kept in a humidified atmosphere of 95% air and 5% CO_2_ at 37 °C and were passaged twice weekly.

### 4.5. Immuno-Fluorescence Microscopy 

In order to evaluate the internalisation property of FA-conjugated tHBcAg VLNPs into HeLa cells, anti-tHBcAg serum was used to detect the internalised tHBcAg particles. The cells (1.0 × 10^5^ cell/mL) were sub-cultured in a six-well plate as described by Biabanikhankahdani et al. [28]. After washing the cells with FA-depleted RPMI1640 medium, FA-conjugated tHBcAg VLNPs (25 μg/mL in 1 mL medium) were added to each well. The cells were kept at 37 °C as described by Biabanikhankahdani et al. [28]. In this experiment, the rabbit anti-tHBcAg serum (1:200 dilution) and the Alexa Fluor 488 conjugated goat anti-rabbit IgG antibody (1:1000 dilution) were used as the primary and secondary antibodies, respectively. The cells were viewed under an Olympus fluorescence microscope (Live Cell Imaging, Center Valley, PA, USA). Untreated cells and cells treated with tHBcAg VLNPs served as controls. 

### 4.6. Conjugation of DOX to tHBcAg VLNPs

The carboxylate groups of the tHBcAg VLNPs were activated by EDC and Sulfo-NHS; then, the activated tHBcAg VLNPs were combined with DOX. tHBcAg VLNPs in 100 mM sodium phosphate buffer (pH 6.0) were incubated with freshly prepared solution of Sulfo-NHS (2000 M in excess) and EDC (1000 M in excess) at room temperature for 3 h with gentle stirring. After adjusting the pH of solution to 7.4, DOX•HCl (2000 M in excess) and DMSO (20% *v*/*v*) were then added. The mixture was stirred for further 16 h at 4 °C and then applied on a sucrose density gradient (8%–40%) to purify DOX-conjugated tHBcAg VLNPs. The amount of DOX in each fraction (400 µL per fraction) was measured at A_495_, and the amount of protein was determined with the Bradford assay [68].

Protein fractions containing the highest amount of DOX-conjugated VLNPs were dialysed against 25 mM sodium phosphate buffer (pH 7.4) containing 150 mM NaCl (1 L, three times) at 4 °C, and concentrated with VIVASPIN 6. The amount of DOX was quantified based on its extinction coefficient (8030 cm^−1^ M^−1^) and A_495_ value, as described by Zeng et al. [70]. The conjugation efficiency of DOX (CE_DOX_), and the number of conjugated DOX molecules to each nanoparticle (N_DOX_) were calculated with Equations (3) and (4) as follows:

CE_DOX_% = weight_DOX_/weight_tHBcAg particle_ × 100% (3)

N_DOX_ = CE_DOX_ × (Mw_tHBcAg particle_/Mw_DOX_) (4)

where Mw represents the molecular weight. 

DOX-conjugated tHBcAg VLNPs were then further conjugated with FA as described above. The morphology of all tHBcAg samples was examined with TEM.

### 4.7. Native Agarose Gel Electrophoresis (NAGE)

NAGE was used to study the migration profiles of the DOX-conjugated tHBcAg VLNPs according to the method described by Yoon et al. [71]. Proteins were stained with CBB, while DOX bands were observed by ultraviolet (UV) illumination using the Bio-Rad GelDoc 2000 imaging system (Philadelphia, PA, USA).

### 4.8. Transmission Electron Microscopy 

tHBcAg VLNPs and derivatives were absorbed onto carbon-coated grids. Then, the grids were negatively stained with 2% (*w*/*v*) uranyl acetate at room temperature for 5 min and viewed under a Hitachi transmission electron microscope (H7700, Hitachi, Ltd., Tokyo, Japan).

### 4.9. Cellular Uptake of DOX-Conjugated tHBcAg VLNPs

Cancer and normal cells were cultured in six-well plates (1.0 × 10^5^ cells per well), as described by Biabanikhankahdani et al. [28]. Then, free DOX, tHBcAg VLNPs conjugated with DOX (tHBcAg-DOX), and tHBcAg VLNPs conjugated with DOX and FA (FA-tHBcAg-DOX) were added at equivalent DOX concentrations (5 μg/mL). Internalised DOX was measured at A_490_ using a microplate reader (ELX 800; BioTeck Instruments, Winooski, VT, USA). 

### 4.10. Live-Cell Imaging

Cellular uptake of FA-tHBcAg-DOX VLNPs, tHBcAg-DOX VLNPs, and free DOX was evaluated using live cell imaging. HeLa (2.0 × 10^5^ cell/mL), HT29 (2.0 × 10^5^ cell/mL), 3T3 (1.0 × 10^5^ cell/mL) and CCD-112 (1.0 × 10^5^ cell/mL) cells were sub-cultured in six well plates. The old medium was then exchanged with 1 mL fresh medium containing FA-tHBcAg-DOX VLNPs, tHBcAg-DOX VLNPs, and free DOX with the equal DOX concentration (5 µg/mL), as described by Biabanikhankahdani et al. [32]. The cells were fixed and imaged using the Olympus Live Cell Imaging (EX_480_ nm and Em_535_ nm). 

### 4.11. Cytotoxicity of DOX-Conjugated tHBcAg VLNPs

The cytotoxicity of FA-tHBcAg-DOX VLNPs, tHBcAg-DOX VLNPs, and free DOX towards cancer and normal cells was evaluated using the MTT cell viability assay. Cancer cells (HeLa, HT29) and normal cells (3T3, CCD-112) were cultured in 96-well plates (10,000 and 7000 cells/well, respectively). Then, different concentrations of DOX formulations and free DOX (0.01–10 µM) in fresh medium were added to the cells. UV-absorbance at 570 nm was measured using the Uquant Elisa plate reader (BioTeck Instruments, Winooski, VT, USA). The cytotoxicity of tHBcAg VLNPs towards these cells served as a negative control. 

## 5. Conclusions

In summary, this study demonstrated the in vitro efficacy of a dual conjugated drug delivery system derived from tHBcAg VLNPs for anticancer therapy. The tHBcAg VLNPs conjugated with DOX and FA significantly enhanced the cellular uptake and the amount of DOX in cancer HeLa and HT29 cells, leading to enhanced anti-tumour effects. The fabrication of this system is simple, and it has the potential to be easily modified for other combinatorial drug deliveries. Then, these dual bioconjugated VLNPs were compared with pH-responsive VLNPs. Both drug delivery systems have their advantages and limitations, depending on different factors such as the size, charge, molecular weight and solubility of the desired drug. 

## Figures and Tables

**Table 1:** Comparisons of different drug delivery systems based on HBcAg VLNPs.

Parameters		Drug Delivery System	
		Dual Conjugation (FA-tHBcAg-DOX)	pH-Responsive (FA-N-tHBcAg-PAA-DOX)
N_FA_		470	953
N_DOX_		1600	946
Cellular uptake by cancer cells compared to free DOX	HT29	3.29-fold higher	4-fold higher
	HeLa	3.62-fold higher	-
Cellular uptake by normal cells compared to free DOX	CCD-112	2.27-fold lower	1.64-fold lower
	3T3	2.12-fold lower	-
Cytotoxicity towards cancer cells compared to free DOX	HT29	2.04-fold higher	7.21-fold higher
	HeLa	2.13-fold higher	-
Cytotoxicity towards normal cells compared to free DOX	CCD-112	2.47-fold lower	1.84-fold lower
	3T3	2.20-fold lower	-

Note: (FA-tHBcAg-DOX) tHBcAg VLNPs conjugated covalently with folic acid (FA) and DOX; (FA-N-tHBcAg-PAA-DOX) FA-conjugated tHBcAg VLNPs using the nanoglue and loaded with PAA-DOX; N_FA_ = number of FA molecules conjugated to each nanoparticle; N_DOX_ = number of DOX molecules conjugated to or packaged within each nanoparticle; HT29 = human colorectal cancer cell line; HeLa = human cervical cancer cell line; 3T3 = normal fibroblast cells; CCD-112 = colorectal normal cell line.
